# Association between delivery methods for enteral nutrition and physical status among older adults

**DOI:** 10.1186/s40795-019-0318-3

**Published:** 2020-01-14

**Authors:** Tetsuro Hayashi, Masato Matsushima, Hidetaka Wakabayashi, Seiji Bito

**Affiliations:** 1 Division of Clinical Epidemiology, National Hospital Organization Tokyo Medical Center, 2-5-1 Higashigaoka, Meguro-ku, Tokyo, 152-8902 Japan; 2 Division of Clinical Epidemiology, Research Center for Medical Sciences, The Jikei University School of Medicine, Tokyo, Japan; 3 Department of Rehabilitation Medicine, Yokohama City University Medical Center, Yokohama, Japan

**Keywords:** Activities of daily living, Aging, Enteral nutrition, Frailty, Mortality

## Abstract

**Background:**

The physical status of patients who received enteral nutrition is still unclear. We aimed to compare the physical functional status among older adult patients who underwent percutaneous endoscopic gastrostomy (PEG) and those with nasogastric feeding.

**Methods:**

We conducted a retrospective cohort study in an acute care hospital from August 1, 2009 to March 31, 2015. We included older adult patients (age ≥ 65 years) who were administered PEG or nasogastric feeding during hospitalization and received enteral nutrition for ≥14 days. We excluded patients who were completely bedridden at the administration of enteral nutrition. The primary outcome was death or becoming bedridden at discharge. The incidence of being bedridden among the patients who survived and received enteral nutrition at discharge was also compared according to the enteral nutrition method used.

**Results:**

Among the 181 patients who were administered enteral nutrition during hospitalization, 40 patients (22%) died and 66 patients (36%) were bedridden at discharge. The proportions of patients who fully resumed oral intake were 30% in the nasogastric group and 2.3% in the PEG group. The adjusted odds ratios comparing PEG feeding to nasogastric feeding were 0.38 (95% CI, 0.16–0.93) for death or being bedridden and 0.09 (95% CI, 0.02–0.40) for being bedridden among the patients who were receiving enteral nutrition at discharge.

**Conclusions:**

Among older adult patients who were administered enteral nutrition, more than half of these patients died or became bedridden. PEG feeding could be associated with a lower risk of becoming bedridden or death in comparison with nasogastric feeding, although PEG feeding may be offered to the most mobile/ambulatory patients within clinical decision-making. Clinicians should carefully consider the administration and choice of enteral nutrition methods, when considering the prognosis of the patients.

## Background

For patients with swallowing limitations, enteral nutrition is always the first choice in such cases where the patient’s bowel function is still intact [1]. The medical measures for enteral nutrition, which involve the ingestion of formula into the intestinal tract via an enteral tube, primarily involve percutaneous endoscopic gastrostomy (PEG) and nasogastric tube feeding.

A systematic review showed that there was no significant difference between tube feeding techniques in terms of the mortality rate and complications, and PEG feeding is superior to nasogastric feeding in intervention failure and quality of life measures outcomes [2–4]. Therefore, the guidelines recommend that PEG feeding is preferable to nasogastric feeding for the patients who are expected to receive enteral nutrition for longer than several weeks [5, 6].

In Japan, the survival rate of patients after receiving PEG feeding is higher than in Western countries [7–9]. However, substitute decision-makers sometimes feel regret for the choice of PEG feeding, and decision conflict have an influence on their decision regret [10]. Many family members feel that they do not have enough discussions on the choice of enteral feeding methods [11].

Physicians, speech pathologists, and dietitians should have sufficient discussions on enteral nutrition with patients and their families and provide fully information about advantages and disadvantages of enteral feeding methods. By contrast, only a few studies have examined the physical functional status among the patients who were administered enteral nutrition. In this context, we aimed to investigate the physical status of patients following enteral nutrition and to compare the difference between the feeding methods.

## Methods

### Design, setting, and participants

This study involved a retrospective analysis of data from the electronic medical record database of Tokyo Medical Center in Japan, from August 1, 2009 to March 31, 2015. Tokyo Medical Center is an educational acute care hospital with 780 beds, including 30 intensive care units and 50 psychiatric beds; the average length of hospital stay at this center is 12.9 days.

The study included patients aged ≥65 years who were admitted to the study institution and received enteral nutrition for ≥14 days during the hospitalization periods. The exclusion criteria were as follows: patients with prior history of enteral nutrition, patients with severe disturbance of consciousness (comatose; defined by the 3-digit code of 100, 200 or 300 on the Japan Coma Scale [12]), patients with mechanical ventilation (defined by non-invasive positive pressure ventilation and/or invasive mechanical ventilation), patients treated in the intensive care unit and psychiatric care beds, patients who were bedridden at the administration of enteral nutrition, and patients without any available records of physical function. We set a lower limit of the period of receiving enteral nutrition at 14 days in order to focus on patients who required long-term or permanent enteral nutrition, since the guidelines recommend PEG feeding for patients who are expected to receive enteral nutrition for longer than several weeks [5, 6]. Bedridden patients at the administration of enteral nutrition were excluded in order to appropriately clarify the change in physical function. We evaluated the physical function of patients by using the “severity and nursing care needs assessment indicator for the general ward [13, 14].” The Ministry of Health, Labor, and Welfare (MHLW) in Japan has made it mandatory for all Japanese hospitals to use this indicator to assess the severity and nursing care needs of inpatients, and trained nurses evaluate patients by using the indicator. The indicator consists of A and B scores, wherein the A score is related to disease severity and the B score is related to the physical function of patients (Additional file 1: Table S1). The B score is the sum of the grades for each item related to nursing support for daily activities. We defined being bedridden as a B score of 10–12 in this trial, since this score reflects patients with complete dependence.

### Exposure and outcome variables

Patients who received enteral nutrition via a nasogastric tube for ≥14 days and never underwent the PEG procedure were allocated to the nasogastric group. In contrast, patients who started receiving enteral nutrition via PEG feeding or those who started receiving enteral nutrition via a nasogastric tube and subsequently switched to PEG feeding were allocated to the PEG group. Since this study was a retrospective data analysis, this allocation was completely based on the clinical decisions.

Our primary outcome was death or becoming bedridden at discharge. We also performed a sensitivity analysis to assess the patients who became bedridden among those who survived and received enteral nutrition at discharge.

### Potential confounders

We considered the following variables as potential confounders, and analyzed the effect of these confounders on the outcome: age, sex, body mass index (BMI), diagnosis, presence of dementia, comorbidities (based on the updated Charlson comorbidity index [15, 16]), serum albumin level, disease severity, physical function, physical restraints, physical therapy, daily intake of enteral nutrition, estimated energy requirement, and the Geriatric Nutritional Risk Index [17]. Disease severity was estimated based on the A score of the “severity and nursing care needs assessment indicator for the general ward”. The A score quantifies medical care: wound care, blood pressure monitoring, urine volume monitoring, respiratory care, ≥3 intravascular lines, electrocardiogram monitoring, continuous infusion, blood product use, and specialized treatments such as chemotherapy. We defined patients with an A score of ≥2 as severe patients in accordance with the MHLW definition. We also defined patients with a B score of 7–9 as moderately dependent based on their physical function, whereas a B score of 0–6 means less dependent. The use of physical restraints was evaluated in cases where physical restraints were applied in patients, including a wide cloth bandage across the trunk or on ≥1 limb. The use of physical therapy was evaluated in cases where patients received rehabilitation by physical therapists. Estimated energy requirement was calculated, using the Harris-Benedict equation and adjusting this value by activity factor (AF) and stress factor (SF) [18, 19]. The values of AF were 1.2 for patients who did not receive physical therapy and 1.3 for patients who received physical therapy. The values of SF were 1.0 for patients diagnosed as stroke/ neurological diseases and 1.2 for others.

### Data collection

Patient data were acquired from the institutional electronic medical record database and were automatically extracted by an institutional system manager who did not participate in the analysis. Only the information regarding prior history of enteral nutrition, daily intake of enteral nutrition, and decision-making process was assessed by confirming the record of each patient.

### Statistical methods

We used the Wilcoxon rank sum test for continuous variables. A bivariate analysis between the two groups was conducted using the Chi-squared test or Fisher’s exact test based on the number of samples. We used multiple imputation by chained equation to compensate for missing values. We fit logistic regression models for the outcome, adjusting for potential confounders. The *P* values were two-tailed, and *P* < 0.05 was considered significant. All the analyses were conducted using STATA 12 software (StataCorp, College Station, TX, USA).

## Results

**Fig. 1:**
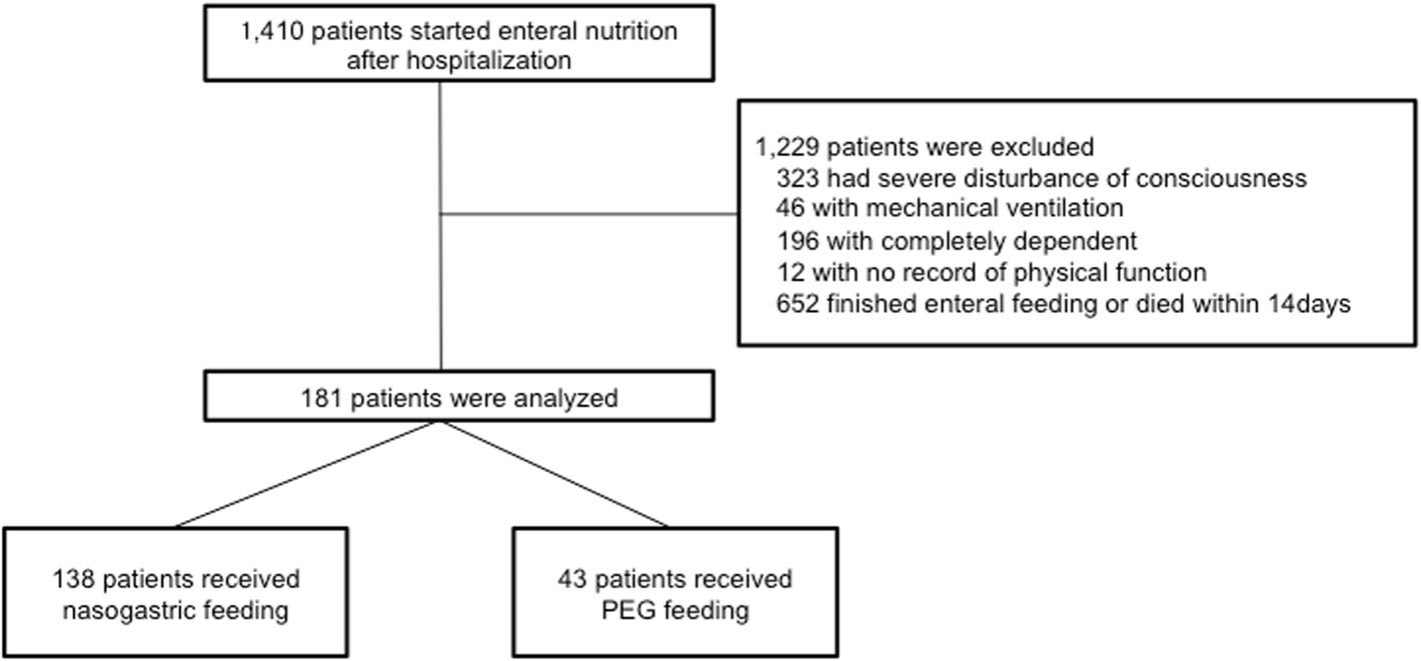
Study flow diagram. Abbreviation: PEG, percutaneous endoscopic gastrostomy

We identified 1410 patients who started receiving enteral nutrition during their hospitalization period. Based on the eligibility criteria, 181 patients were finally enrolled, including 138 patients in the nasogastric group and 43 patients in the PEG group (Fig. 1). All the patients in the PEG group had received prior enteral nutrition via a nasogastric tube and subsequently underwent PEG after a median (interquartile range, IQR) interval of 45 (26–70) days.

The percentages of the documented discussions for the choice of enteral feeding methods between physicians, patients and their families were 54% in the nasogastric group and 93% in the PEG group. The discussions were held after physicians received assessment reports of patients’ swallowing function from their speech pathologists, dietitians and/or rehabilitation physicians. The major reason of choosing long-term nasogastric tube feeding was the families’ preference of the nasogastric tube method to PEG method.

Table 1 shows the characteristics of the patients in the nasogastric group and PEG group. The patients in the nasogastric group had a poor physical functional status at baseline in comparison with the patients in the PEG group. The median (IQR) length of hospital stay in the overall cohort was 84 (56–125) days, and was shorter in the nasogastric group than in the PEG group (78 days [52–123] vs. 95 days [61–151], respectively; *P* = 0.04). There were one patient with leg fracture and five patients with head and neck cancer in the population. The numbers of patients who received therapy treatments from speech pathologists were 63 (46%) in NGT group and 17 (40%) in PEG group. The percentages of average intake to the estimated energy requirements were 69% in NGT group and 70% in PEG group.

**Table 1:** Demographic characteristics of the patients

	Nasogastric (*n* = 138)	PEG (*n* = 43)	*P*
Age (years)	82 [78, 87]	84 [76, 88]	0.78
Female	55 (39.9)	17 (39.5)	0.97
BMI < 18.5^a^	32 (35.2)	14 (45.2)	0.32
Diagnosis			
Stroke/neurological diseases	61 (44.2)	24 (55.8)	0.09
Respiratory diseases	27 (19.6)	12 (27.9)	
Malignancy	21 (15.2)	4 (9.3)	
Others	29 (21.0)	3 (7.0)	
Dementia	26 (18.8)	6 (14.0)	0.46
Updated Charlson Comorbidity Index			
0–2	122 (88.4)	39 (90.7)	0.44
3–5	10 (7.3)	1 (2.3)	
≥ 6	6 (4.4)	3 (7.0)	
Serum albumin (mg/dL) ^b^			
< 2.5	31 (22.5)	4 (9.3)	0.06
Severity (A score)^c^			
< 2	67 (48.6)	20 (46.5)	0.82
≥ 2	71 (51.5)	23 (53.5)	
Physical function (B score) ^d^			
< 7	17 (12.3)	13 (30.2)	< 0.01^*^
7–9	121 (87.7)	30 (69.8)	
Received physical restraints	86 (62.3)	27 (62.8)	0.96
Received physical therapy	133 (96.4)	40 (93.0)	0.40
Daily intake of enteral nutrition (kcal)	1065 [809, 1200]	900 [645, 1107]	0.03^*^
Estimated energy requirement (kcal)^a^	1536 [1320, 1714]	1284 [1135, 1699]	< 0.01^*^
Geriatric nutritional risk index^e^	80 [71, 87]	80 [75, 85]	0.78

Data are presented as number (%) except for age, daily intake of enteral nutrition, estimated energy requirement, and Geriatric nutritional risk index, which are presented as median [interquartile range]

*P < 0.05

^a^33% missing

^b^5% missing

^c^A score of ≥2 means severe disease

^d^B score of 7–9 means moderately dependent, whereas B score of 0-6 means less dependent

^e^35% missing

The outcome measures are shown in Table 2. Among the 181 patients who were administered enteral nutrition during hospitalization, 40 patients (22%) died and 66 patients (36%) were bedridden at discharge. The number of patients who were dead or bedridden at discharge was 84 patients (61%) in the nasogastric group and 22 patients (51%) in the PEG group. Among the patients who resumed oral intake, 23 patients (53%) received therapy treatment by speech pathologists. Of 98 patients who continued to receive enteral nutrition at discharge, the number of patients who became bedridden was 43 patients (67.2%) in the nasogastric group and 14 patients (41.2%) in the PEG group. The proportions of patients who fully resumed oral intake were 30% in the nasogastric group and 2.3% in the PEG group. There were two patients with temporal PEG feeding after surgery of head and neck cancer. All the patients who could not resume oral intake or who could not continue with enteral nutrition eventually died during the hospitalization period. The number of patients who were discharged to home was 40 patients (22%). There was no difference in the incidence of aspiration pneumonia.

**Table 2:** Outcome measures

	Nasogastric (*n* = 138)	PEG (*n* = 43)	*P*
Dead and bedridden	84 (60.9)	22 (51.2)	0.26
Dead	32 (23.2)	8 (18.6)	0.53
Bedridden	52 (37.7)	14 (32.6)	0.54
Oral intake resumption	42 (30.4)	1 (2.3)	< 0.001^*^
Receiving enteral nutrition at discharge	64 (46.4)	34 (79.1)	< 0.001^*^
Discharge disposition			
Home	25 (18.1)	15 (34.9)	0.02*
Nursing facilities	9 (6.5)	5 (11.6)	0.33
Other hospitals	72 (52.2)	15 (34.9)	0.048^*^
Complication			
Aspiration pneumonia	17 (12.3)	7 (16.3)	0.50

Data are presented as number (%)

^*^P < 0.05

Death or being bedridden at discharge was used as the outcome for the logistic regression analysis, which was adjusted for potential confounders (Table 3). The analysis showed that, compared with the nasogastric group, the PEG group had a significantly lower risk of death or being bedridden at discharge. Of 98 patients who survived and continued to receive enteral nutrition at discharge, the logistic analysis for being bedridden indicated a decreased risk in the PEG group. The higher daily intake of enteral nutrition was associated with the better outcome of physical status.

**Table 3:** Logistic regression analysis for death and becoming bedridden

Predictor variables	Death or bedridden^a^	Death^a^	Bedridden^b^
	OR (95% CI)	OR (95% CI)	OR (95% CI)
PEG feeding (nasogastric feeding as reference)	0.38 (0.16–0.93)^*^	0.56 (0.19–1.64)	0.09 (0.02–0.40)^*^
Age (years)	1.01 (0.95–1.07)	1.03 (0.95–1.11)	1.02 (0.93–1.12)
Female (male as reference)	1.36 (0.59–3.12)	0.30 (0.10–0.88)^*^	3.90 (0.75–20.19)
BMI < 18.5 (≥18.5 as reference)	0.78 (0.20–3.01)	1.82 (0.41–8.00)	0.89 (0.10–8.34)
Diagnosis (stroke/neurological diseases as reference)			
Respiratory diseases	2.91 (0.73–11.64)	0.80 (0.17–3.88)	2.21 (0.28–17.37)
Malignancy	0.59 (0.13–2.76)	0.79 (0.13–4.89)	2.44 (0.17–35.72)
Others	0.35 (0.09–1.38)	0.34 (0.06–1.90)	0.24 (0.02–2.99)
Dementia	0.35 (0.13–0.97)^*^	0.84 (0.25–2.90)	0.59 (0.11–3.21)
Updated Charlson Comorbidity Index (score of 0–2 as reference)			
3–5	0.65 (0.14–3.05)	0.35 (0.04–2.82)	0.31 (0.02–4.69)
≥ 6	0.48 (0.08–2.97)	0.82 (0.09–7.39)	0.03 (0.00–1.09)
Albumin < 2.5 mg/dL (level ≥ 2.5 mg/dL as reference)	0.88 (0.26–2.92)	1.78 (0.45–7.11)	0.27 (0.02–2.88)
Severity: A score ≥ 2 (score of 0–1 as reference)^c^	1.05 (0.49–2.26)	0.64 (0.26–1.54)	3.31 (0.76–14.36)
Physical function: B score = 7–9 (score of 0–6 as reference)^d^	1.77 (0.68–4.64)	1.36 (0.39–4.66)	1.34 (0.27–6.80)
Physical restraints (no physical restraints as reference)	1.69 (0.79–3.65)	1.53 (0.56–4.15)	5.08 (1.19–21.71)^*^
Physical therapy (no physical therapy as reference)	0.44 (0.08–2.56)	0.12 (0.01–0.95)^*^	0.15 (0.00–12.55)
Daily intake of enteral nutrition (kcal/100)	0.77 (0.68–0.88)^*^	0.76 (0.66–0.88)^*^	0.66 (0.52–0.85)^*^
Estimated energy requirement (kcal/100)	1.13 (0.83–1.53)	1.15 (0.85–1.56)	1.38 (0.79–2.40)
Geriatric nutritional risk index	0.98 (0.93–1.03)	0.98 (0.91–1.05)	0.97 (0.86–1.09)

^*^P < 0.05

^a^181 patients were analyzed

^b^98 patients who survived and continued to receive enteral nutrition at discharge were analyzed

^c^A score of ≥2 means severe disease

^d^B score of 7–9 means moderately dependent, whereas B score of 0–-6 means less dependent

## Discussion

The results of this study indicate that PEG feeding is preferable to nasogastric feeding in terms of the patients’ physical status. We found that patients with PEG feeding have a significantly lower risk of death or being bedridden. Moreover, among the patients who were receiving enteral nutrition at discharge, those with PEG feeding had a significantly lower risk of being bedridden. The reasons for the better outcomes in the PEG group remain unclear, although they may be explained by the fact that PEG feeding was associated with reduced tube troubles and hence enabled steady nutrition consumption [3, 20]. Moreover, the degree and period of physical restraints or physical therapy might affect the outcomes, since we only assessed the presence or absence of physical restraints and physical therapy.

Patients in the nasogastric group were likely to take higher daily intake of enteral nutrition. The underlying reason may be explained that patients in the nasogastric group were likely to have higher estimated energy requirements. After the adjustment, the higher daily intake of enteral nutrition was associated with the better physical status.

One interesting finding was that the patients in the nasogastric group had a higher probability of resuming oral intake at discharge, despite the fact that they had a severe condition at baseline. This result may indicate that there were other factors that predict the probability of resuming oral intake, which could not be explored in this study. Alternatively, this may be explained by the fact that patients who were not able to consume an oral diet eventually received the PEG procedure.

There was no significant difference in the presence of dementia between NGT and PEG groups. The overall prevalence of dementia was two out of ten in our analysis, and the frequency of dementia may be underestimated, since the sensitivity and specificity of detecting dementia in our database were 37.5 and 100% [21]. In our analysis, the presence of dementia was associated with decreased risk of death and bedridden, although a previous systematic review shows that the association between presence of dementia and mortality among patients with enteral nutrition remains controversial [22].

We found that more than half of the patients in each group underwent physical restraints during their hospitalization period. The application of physical restraints could possibly lead to further complications [23]. In contrast, medical staff tended to use physical restraints for patients with tube feeding in order to prevent tube removal [24]. Attanasio noted that tube replacement is more frequent with nasogastric feeding than with PEG feeding, and it may be a cause of physical restraints [25], although there was no significant difference in the frequency of physical restraint use between the groups in our study. It is important to minimalize the use of physical restraints for the patients with enteral nutrition.

The study limitation includes that the allocation of PEG or nasogastric groups was completely based on the clinical decisions, and PEG feeding may be offered to the most mobile/ambulatory patients. We included potential confounders to adjust for the final model. However, there could be residual confounding, since our study was a retrospective study and PEG feeding would be considered for patients with better prognosis during the hospital stay. In addition, we could not determine Cronbach’s alpha and the test-retest reliability correlations of the “severity and nursing care needs assessment indicator for the general ward,” although previous studies have confirmed its concurrent validity and criterion validity [13, 14]. Finally, the dataset had also limited information on the proportion of food/fluid balance and protein intake.

## Conclusion

Among older adult patients who were administered enteral nutrition, more than half of these patients died or became bedridden. PEG feeding could be associated with a lower risk of becoming bedridden or death in comparison with nasogastric feeding, although PEG feeding may be offered to the most mobile/ambulatory patients within clinical decision-making. Clinicians should carefully consider the administration and choice of enteral nutrition methods, when considering the prognosis of physical status of the patients. We believe that these findings may be useful for decision making by patients and clinicians.

## Supplementary information

**Additional file 1: Table S1** The B score of the “Severity and nursing care needs assessment indicator for general ward”. The B score of the “Severity and nursing care needs assessment indicator for general ward” is related to the physical functional status of inpatients. It comprises various items related to nursing support for daily activities.

## Data Availability

The datasets used and analyzed during the current study are not publicly available due to the patients’ privacy but are available from the corresponding author on reasonable request.

## Abbreviations

PEG: Percutaneous endoscopic gastrostomy
