# Supplementary material for: Association between delivery methods for enteral nutrition and physical status among older adults
Source: BMC Nutr. 2020 Jan 14;6:2. doi: 10.1186/s40795-019-0318-3 (PMC7050869; doi:10.1186/s40795-019-0318-3)
Supplement: Supplementary file 1 — Additional file 1: Table S1 The B score of the “Severity and nursing care needs assessment indicator for general ward”. The B score of the “Severity and nursing care needs assessment indicator for general ward” is related to the physical functional status of inpatients. It comprises various items related to nursing support for daily activities. [file 40795_2019_318_MOESM1_ESM.docx]

**Table S1. The B score of the “Severity and nursing care needs assessment indicator for general ward”.**

| Daily activity | Grade |  |  |
| --- | --- | --- | --- |
|  | 0 | 1 | 2 |
| Rolling over | Independent | Needs some help | Completely dependent |
| Getting up | Independent | Completely dependent | NA |
| Sitting | Independent | Needs some help | Completely dependent |
| Transfers | Independent | Needs some help | Completely dependent |
| Oral care | Independent | Completely dependent | NA |
| Eating | Independent | Needs minor assistance | Needs major assistance |
| Dressing | Independent | Needs minor assistance | Needs major assistance |

Abbreviation: NA, not applicable

The B score of the “Severity and nursing care needs assessment indicator for general ward” is related to the physical functional status of inpatients. It comprises various items related to nursing support for daily activities.
